# Resolving Salt-Induced Agglomeration of Laponite Suspensions Using X-ray Photon Correlation Spectroscopy and Molecular Dynamics Simulations

**DOI:** 10.3390/ma16010101

**Published:** 2022-12-22

**Authors:** Sohaib Mohammed, Meishen Liu, Qingteng Zhang, Suresh Narayanan, Fan Zhang, Greeshma Gadikota

**Affiliations:** 1 School of Civil and Environmental Engineering, Cornell University, Ithaca, NY 14853, USA; 2 X-ray Science Division, Advanced Photon Source, Argonne National Laboratory, Lemont, IL 60439, USA; 3 Materials Measurement Science Division, National Institute of Standards and Technology, Gaithersburg, MD 20899, USA

**Keywords:** laponite, CaCl_2_, MgCl_2_, CsCl, XPCS, MD, self-assembly, gel formation

## Abstract

Linking the physics of the relaxation behavior of viscoelastic fluids as they form arrested gel states to the underlying chemical changes is essential for developing predictive controls on the properties of the suspensions. In this study, 3 wt.% laponite suspensions are studied as model systems to probe the influence of salt-induced relaxation behavior arising from the assembly of laponite nanodisks. X-ray Photon Correlation Spectroscopy (XPCS) measurements show that laponite suspensions prepared in the presence of 5 mM concentrations of CaCl_2_, MgCl_2_ and CsCl salts accelerate the formation of arrested gel states, with CaCl_2_ having a significant impact followed by CsCl and MgCl_2_ salts. The competing effects of ion size and charge on relaxation behavior are noted. For example, the relaxation times of laponite suspensions in the presence of Mg^2+^ ions are slower compared to Cs+ ions despite the higher charge, suggesting that cation size dominates in this scenario. The faster relaxation behavior of laponite suspensions in the presence of Ca^2+^ ions compared to Cs^+^ ions shows that a higher charge dominates the size of the ion. The trends in relaxation behavior are consistent with the cluster formation behavior of laponite suspensions and the electrostatic interactions predicted from MD simulations. Charge balance is achieved by the intercalation of the cations at the negatively charged surfaces of laponite suspensions. These studies show that the arrested gel state of laponite suspensions is accelerated in the presence of salts, with ion sizes and charges having a competing effect on relaxation behavior.

## 1. Introduction

Clay suspensions have been widely incorporated in the enhanced oil recovery industry from conventional and unconventional reservoirs as hydrogels, drilling fluids and surface-active materials due to their nanoscopic size, anisotropic shape, nontoxic nature and low cost [1]. Laponite, a synthetic smectite clay, and its suspensions have been examined as a hydrogel to mitigate the excess water production and improve the hydrocarbon production from mature reservoirs [2]. The incorporation of laponite clays in hydrogel compositions have overcome the mechanical, thermal and elasticity limitations of conventional gels and improved the gel performance under adverse reservoir conditions [2].

Laponite particles have also been used to stabilize oil–water emulsions and proved a promising emulsification potential due to its high purity and uniform shape [3]. The performance of laponite as an emulsion stabilizer is affected by the laponite concentrations and emulsion compositions [4,5]. For example, the presence of salt in the aqueous phase directly influences the behavior of laponite particles at the oil–water interface and consequently alters the emulsion stabilization potential of laponites. At a sufficiently high concentration of salts, laponite particles coagulate and their partitioning at the oil–water interface enhances [6].

A fundamental understanding of the influence of salts on laponite relaxation behavior or aging dynamics has significant scientific and technological implications in the enhanced oil recovery applications. The influence of salt concentrations on the relaxation behavior of laponite has been shown to be non-monotonic [7]. For instance, studies with barium chloride salts showed that at low and high electrolyte concentrations, the slow aggregation of laponite to yield densely packed aggregates is noted. At intermediate salt concentrations, however, the double layer collapses and rapid aggregation is observed [8]. Prior studies showed that monovalent and divalent salts such as Na^+^ and Ca^2+^ at different concentrations accelerate the aging dynamics of the laponite suspensions [9,10,11,12].

In addition to the salt effect, the rheological properties of aqueous laponite suspensions are strongly dependent on laponite concentrations and on the ionic strength of the medium due to the competition between the short-range van der Waals attraction and long-range electrostatic attraction or repulsion arising from the shape and charge anisotropy of laponite particles [13]. In this context, the relaxation of suspended laponites is found to evolve slowly and is usually characterized by two processes: (a) fast relaxation (cage diffusion) associated with the rapid motion of particles inside the cage formed by its neighbors, followed by (b) the slow structural rearrangement of the formed cages, which is the relaxation of the whole suspension [14].

Based on these insights, the phase diagram of laponite suspensions was developed to represent the formation of various phases including isotropic liquid, isotropic gel, nematic gel and flocculation at varying laponite concentrations and ionic strength of the associated medium [15,16,17,18,19]. The isotropic liquid phase is identified by low ionic strength and laponite concentrations lower than 1.5 wt%; isotropic and nematic gels are obtained at laponite concentrations of about 2–3 wt% and ≥3 wt%, respectively, while the flocculation phase is found at high ionic strength for all laponite concentrations. Two microstructures have been proposed for the laponite particles in the arrested state [20]. The first structure is characterized by the formation of a laponite particle network connected by the positive rim and the negative surface, which results in an attractive gel structure. The second structure is a repulsive glass in which the laponite particles are uniformly dispersed due to the repulsive environment that prevents the particles from aggregating with each other. At laponite concentrations < 2.4 wt% and concentrations > 2.4 wt%, attractive gel structures and repulsive glass structures of laponite are formed, respectively [17,21]. The dispersions follow different routes to reach arrested states depending on the concentration and composition of the laponite suspensions [22,23]. When studying laponite relaxation behavior, aging refers to the evolution of physical properties with a waiting time [24].

These insights and the established phase diagrams have been resolved using several experimental and computational approaches such as Dynamic Light Scattering (DLS) [14], X-ray Photon Correlation Spectroscopy (XPCS) [25,26], Small Angle X-ray Scattering (SAXS) [27], Molecular Dynamics (MD) simulations [14], Brownian Dynamics Simulations [28,29], and Monte Carlo simulations [30]. In addition to the ionic strength and the laponite concentrations, other factors influencing the structure and dynamics of laponite suspensions include the aging time, temperature, and hydration [7,31,32,33]. In this context, simulation studies demonstrated that the microstructural and dynamical properties in the relaxation process and in the arrested states depend on the number density and charge distribution on the edges and faces [28,29,34,35,36].

While prior studies provide extensive insights into the relaxation behavior of laponite suspensions, limited organized efforts describing the observed relaxation behavior of laponite in terms of the underlying energetic interaction exist. To address this challenge, we use XPCS measurements and MD simulations to investigate the relaxation behavior of laponite in the presence of monovalent and divalent salts such as CaCl_2_, MgCl_2_ and CsCl at concentrations of 5 mM in aqueous suspensions bearing 3 wt% laponite. The hypothesis that a higher charge and larger size of ions accelerate relaxation dynamics is explored. Specifically, the competing effects of ions on the relaxation behavior of laponite suspensions are probed. In this context, the spatiotemporal evolution in the relaxation behavior of laponite suspensions from XPCS measurements is interpreted from the perspective of the interaction energies and the organization of laponite molecules.

## 2. Methodology

### 2.1. Sample Preparation

The experiments are performed using laponite powder of XLG grade purchased from BYK Additives & Instruments (certain commercial equipment, instruments, software or materials are identified in this paper to foster understanding. Such identification does not imply recommendation or endorsement by the Department of Commerce or the National Institute of Standards and Technology, nor does it imply that the materials or equipment identified are necessarily the best available for the purpose). The laponite powder is dried at 120 °C for 24 h and the dry powder is slowly dissolved in MilliQ water to make 3 wt.% suspensions. The laponite solution is stirred at 800 rpm for 1 h at an ambient temperature to ensure complete suspension and 10 mL of stock solution is transferred to a glass vial and kept undisturbed for 1 h. CaCl_2_, MgCl_2_ and CsCl stock solutions are added to the laponite dispersion to achieve a final concentration of 5 mM. The laponite samples are kept undisturbed for one week before the characterization.

The dimensions of the suspended laponite disks are resolved by measuring and modeling the small-angle X-ray scattering curves of the salt-free suspensions at ambient temperatures. Fresh solutions of 3 wt% laponite nanodisks are prepared at room temperature using 800 rpm stirring for 1 hr. SAXS measurements are then performed on the suspended nanodisks to resolve the average size and dimensions of the particles. The fitting SAXS curve shows that laponite disks have a diameter and thickness of 34.67 ± 4.6 nm and 1.08 ± 0.06 nm, respectively (see Figure S1).

### 2.2. X-ray Photon Correlation Spectroscopy Measurements

**Figure 1:**
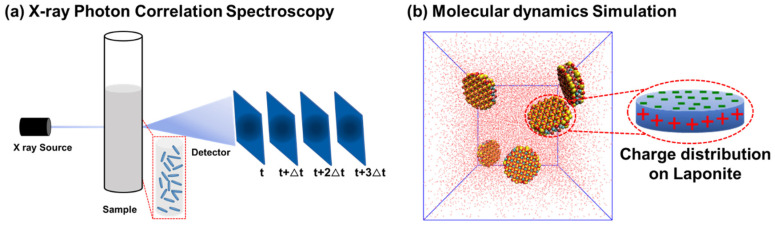
Schematic representation of the experimental setup for X-ray Photon Correlation Spectroscopy (XPCS) measurements for the laponite suspensions is shown in (**a**). Snapshot of the simulated initial configuration showing the laponite nanoparticles dissolved in water molecules and schematics illustrating the charge distribution on laponite particles shown in (**b**). Laponite nanoparticles are shown in VDW representation mode while water molecules are shown in Lines representation mode implemented in VMD software. Water molecules and laponite nanoparticles are initially randomly distributed in the simulation cell to avoid any bias in the following equilibration simulation.

The relaxation behavior of the prepared laponite samples is determined using XPCS measurements [26,37,38] (see Figure 1a) at Sector 8 in the Advanced Photon Source (APS) at Argonne National Laboratory (ANL). An incident beam of 10.9 keV photon energy is used and the scattered intensity is determined by the Large Area Medipix Based Detector Array (LAMBDA) detector. The ensemble-averaged intensity autocorrelation functions, *g*_2_(*Q*, *t*) are calculated using the following expression [39].

(1) $$g_{2}(Q,t)=\frac{{\langle {\langle I_{i,j}\left(t^{\prime }\right)I_{i,j}\left(t^{\prime }+t\right)\rangle }_{t^{\prime }}\rangle }_{i,j}}{{\langle {\langle I_{i,j}\left(t^{\prime }\right)\rangle }_{t^{\prime }}\rangle }_{i,j}{\langle {\langle I_{i,j}\left(t^{\prime }+t\right)\rangle }_{t^{\prime }+t}\rangle }_{i,j}}$$

In this expression, *I*_*i*,*j*_(*t*′) and *I*_*i*,*j*_(*t*′ + *t*) are the scattering intensity collected at pixel (*i*, *j*) and experimental time *t*′ and *t*′ + *t*, respectively. *Q* = 4*π*/*λ* *sin*(*θ*) is the reciprocal space vector of pixel (*i*, *j*), where 2*θ* is the scattering angle of pixel (*i*, *j*) and *λ* is the X-ray wavelength. The pixel average <…>_*i*,*j*_ is performed azimuthally over a narrow band of Δ*Q* with a width of approximately 1.0 × 10^−3^ Å^−1^ to improve the statistics of *g*_2_. The ensemble-averaged intensity autocorrelation function, *g*_2_(*Q*, *t*), is related to the physics of the relaxation behavior through the expression represented below [40].

(2) $$g_{2}(Q,t)-1=C_{}\;exp\left(-2{\left(\frac{t}{\tau_{Q}}\right)}^{\beta_{Q}}\right)$$

In this expression, *C* and $\tau_{Q}$ represent the contrast and characteristic relaxation time, respectively. $\beta_{Q}$ is the Kohlrausch exponent, which is related to the distribution width of the relaxation time, and f(Q,t) is the intermediate scattering function. The dynamics of the laponite suspensions are characterized by monitoring the relaxation time and Kohlrausch exponent obtained from fitting Equation (2) [41].

### 2.3. Molecular Dynamics Simulations

**Initial configurations.** The initial configuration of the simulated laponite suspensions is composed of five nanodisks with a diameter and thickness of 30 Å and 9.2 Å, respectively (see Figure 1b). Although the simulated nanodisk diameter is lower than that of the experimental powder, the atomic structure of the simulated disks exactly mimics the structure of the experimental powder; thus, simulating smaller disks provides the advantage of probing intermolecular interactions with a lower computational cost. Laponite disks are cleaved from a unit cell composed of octahedral magnesium ions sandwiched between two layers of tetrahedral silicon atoms [42,43]. The concentration of laponite disks dissolved in the water molecules is 3 wt% to match the experimental concentration used in this study. The initial configuration is replicated four times to investigate the assembly of laponite disks in water and 5 mM of CaCl_2_, MgCl_2_ and CsCl. The laponite disks and water molecules are modeled using ClayFF [44] and TIP4P [45] force fields, respectively. The 100 Å × 100 Å × 100 Å simulation cells are periodic in x, y and z directions.

**Simulation Algorithm.** Energy minimization on the initial configurations is performed using the steepest descent method to eliminate the steric clashes and inappropriate geometry. MD simulations are then conducted on the optimized configurations for 40 ns in the NVT ensembles at 298 K using a Nose–Hoover thermostat [46,47]. Intramolecular potentials account for bond stretching, angle bending and dihedrals, while intermolecular potentials account for van der Waals and electrostatic interactions, modeled by 12-6 Lennard-Jones and Coulomb’s functions, respectively, as follows:

(3) $$U_{ij}=\sum_{i<j}\left[4\varepsilon_{ij}\left[{\left(\frac{\sigma_{ij}}{r_{ij}}\right)}^{12}-{\left(\frac{\sigma_{ij}}{r_{ij}}\right)}^{6}\right]+\frac{q_{i}q_{j}}{r_{ij}}\right]$$

where $q_{i}$ and $q_{j}$ are the partial charges of atoms *i* and *j* in Coulombs, $r_{ij}$ is the distance between *i* and *j*. LJ parameters (i.e., $\varepsilon_{ij}$ and $\sigma_{ij}$), unlike molecular interactions, were calculated based on the Lorentz–Berthelot combining rules, as follows:

(4) $$\sigma_{ij}=\frac{\sigma_{ii}+\sigma_{jj}}{2}$$

(5) $$\varepsilon_{ij}=\sqrt{\varepsilon_{ii}\varepsilon_{jj}}$$

A cutoff of 14 Å was used to calculate the short-range intermolecular interactions, while long-range electrostatic interactions were treated using Particle Mesh Ewald (PME) [48]. MD runs were performed using GROMACS 5.2 code [49]. The error bars of the data represent standard deviations based on simulations performed in triplicate.

## 3. Results and Discussion

### 3.1. Determination of Laponite Relaxation Behavior Using XPCS Measurements

**Figure 2:**
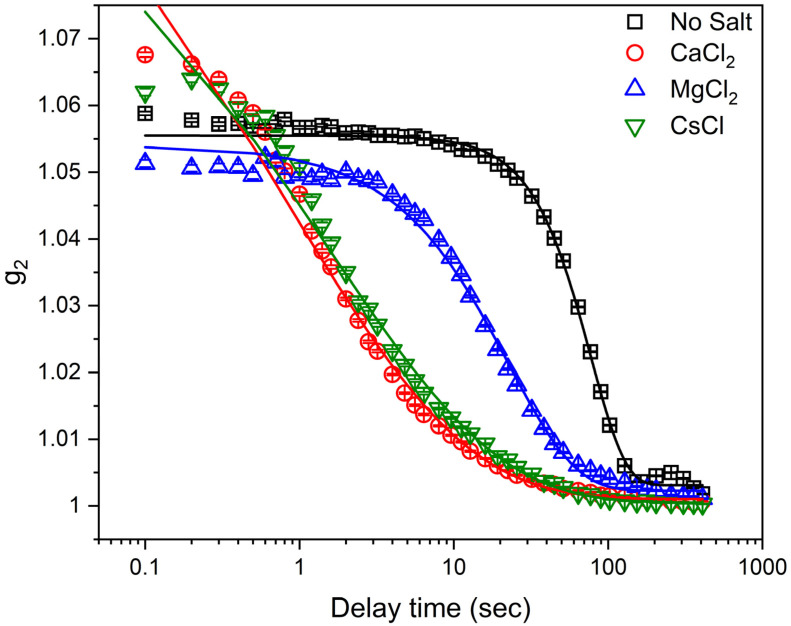
The intensity autocorrelation functions [*g*_2_*(Q,t)*] determined from X-ray Photon Correlation Spectroscopy (XPCS) measurements of 3 wt% aqueous laponite suspension prepared in 5 mM of CaCl_2_, MgCl_2_ and CsCl, and without salt at a *Q* value of 0.01 Å^−1^.

**Figure 3:**
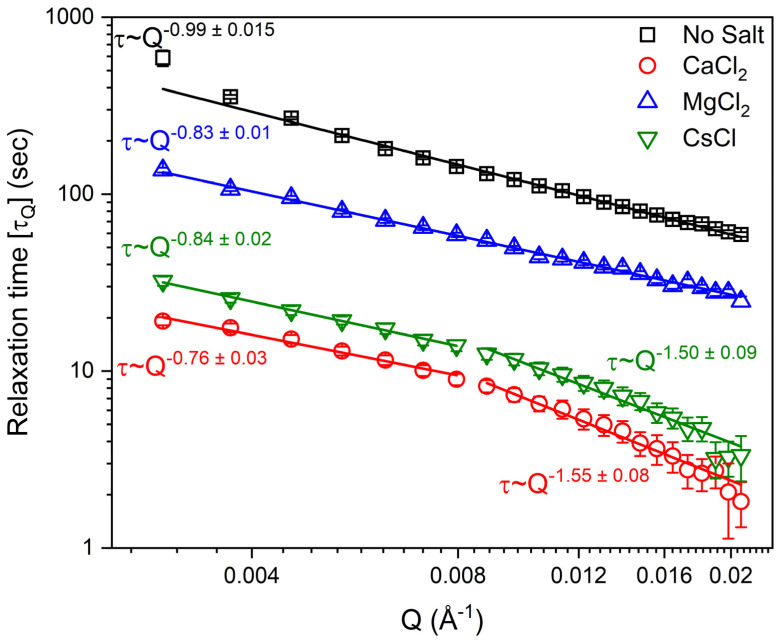
The relaxation time of 3 wt% aqueous laponite suspension prepared in 5 mM of CaCl_2_, MgCl_2_ and CsCl, and without salt as a function of the wave vector, *Q*.

The relaxation behavior of laponite suspensions is determined from the intensity autocorrelation functions, *g*_2_(*Q*, *t*), obtained from XPCS measurements. *g*_2_ of laponite solutions prepared in water and in solutions bearing 5 mM CaCl_2_, MgCl_2_ and CsCl solutions at *Q* = 0.01 Å^−1^ is shown in Figure 2. Acceleration of the relaxation dynamics of laponite suspensions in the presence of salts is evident from the reduced decay time and is in qualitative agreement with previous studies [10,11,50]. Further, the added salts reduced the decay time in the order of CaCl_2_ > CsCl > MgCl_2_, implying that the size and charges of cations influence the dynamics of the suspensions. The relaxation time ($\tau_{Q}$) and the Kohlrausch exponent (*β_Q_*) are extracted from fitting *g*_2_ using Equation (2). The *Q* dependence of the relaxation time ($\tau_{Q}$) (Figure 3) shows that the salt-bearing laponite suspensions have faster relaxation times compared to the salt-free suspensions. Relaxation times for salt-free suspensions are the highest, followed by suspensions prepared in the presence of MgCl_2_, CsCl and CaCl_2_ salts. Further, the relaxation time dependence on *Q* is *τ*_*Q*_ ~ *Q*^−0.99±0.015^ and *τ*_*Q*_ ~ *Q*^−0.83±0.01^ in salt-free and MgCl_2_-bearing suspensions, respectively, which agrees with the dynamics of several colloidal gels [51,52,53]. On the other hand, a transition from *τ*_*Q*_ ~ *Q*^−0.76±0.03^ to *τ*_*Q*_ ~ *Q*^−1.55±0.08^ and from *τ*_*Q*_ ~ *Q*^−0.84±0.02^ to *τ*_*Q*_ ~ *Q*^−1.50±0.09^ was observed in the CaCl_2_- and CsCl-bearing suspensions at *Q* = 0.008 Å^−1^. This transition indicates the different dynamical behavior of laponite aggregates and individual disks in CaCl_2_- and CsCl-bearing suspensions. The *Q*^−1^ relaxation time dependency on the wave vector can be explained by the ultraslow motion of laponite nanoparticles under the action of internal stress, while the *Q*^−2^ dependency of the relaxation time indicates diffusive motions of the scatters [54]. The ultraslow motion (*Q*^~−1^) is observed in the salt-free and MgCl_2_-bearing suspensions, while a transition from *Q*^~−1^ to *Q^~−2^* dependency is observed in the CaCl_2_- and CsCl-bearing suspensions, indicating a transition from ultraslow motion to diffusive motion at *Q* values of about 0.008 Å^−1^ (~78 nm), respectively (see Figure 3). This transition might be attributed to the magnitude of the effect induced by the added CaCl_2_ and CsCl on reducing the repulsive energy barriers needed for laponite particles to approach each other [7].

**Figure 4:**
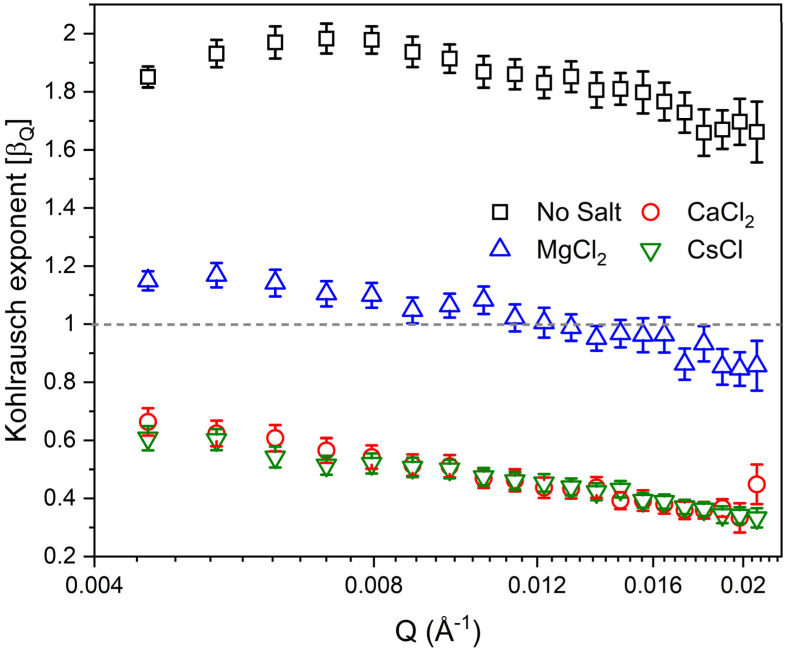
The Kohlrausch exponent of 3 wt% aqueous laponite suspension prepared in 5mM of CaCl_2_, MgCl_2_ and CsCl, and without salt as a function of the wave vector, *Q*.

To better understand the dynamical evolution of salt-free and salt-bearing suspensions, Kohlrausch exponent (*β_Q_*) is obtained from fitting *g*_2_ using Equation (2), which explains the distribution of relaxation times of the laponite suspensions (Figure 4). A value of *β_Q_* < 1 implies that *g*_2_ takes a stretched exponential behavior, suggesting a wide distribution of relaxation times attributed to the existence of dynamical heterogeneities in different spatial scales [55,56]. The stretched behavior of the autocorrelation functions is commonly observed in glass dynamics [41]. A value of *β_Q_* > 1 indicates that the decay of the autocorrelation function takes a compressed, faster-than-exponential behavior that is associated with a hyper-diffusive motion [52,53]. The salt-free suspension shows *β_Q_* > 1 over the probed *Q* range, while MgCl_2_-bearing suspensions are primarily exponential in decay with slight fluctuations in the exponent that are not much higher than the error bar. On the other hand, CaCl_2_- and CsCl incorporated solutions retain *β_Q_* < 1 over the probed *Q* range (see Figure 4).

### 3.2. Determination of Laponite Relaxation Behavior Using MD Simulations

To elucidate the energetic interactions underlying the observed relaxation behavior, molecular dynamics simulations are performed on laponite suspensions prepared with and without salts such as CsCl, MgCl_2_ and CaCl_2_ at concentrations of 5 mM. The aggregation behavior of laponite suspensions was determined from the average size of the self-assembled laponite nanodisks using the expression below:

(6) $$N_{n}=\frac{\sum_{i}i.N_{i}}{\sum_{i}N_{i}}$$

**Figure 5:**
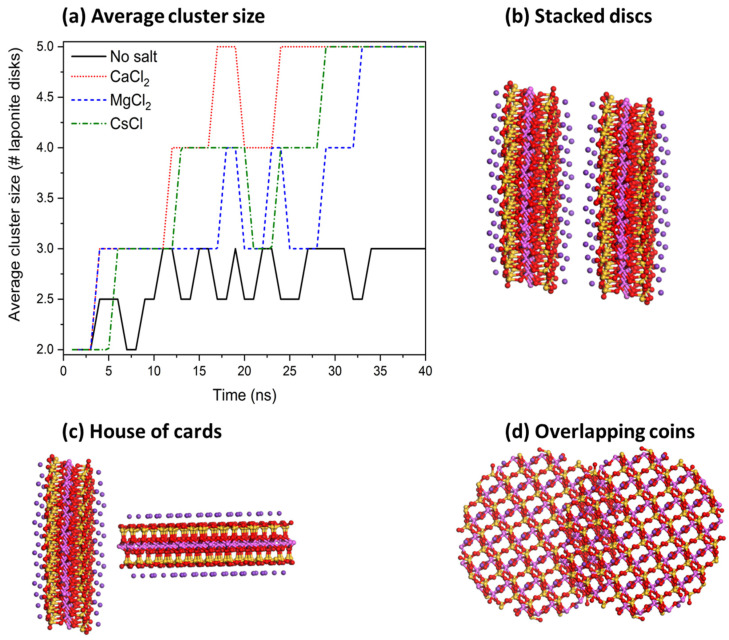
(**a**) The self-assembly profile of the laponite disks as a function of the simulation time in the absence and presence of 5 mM of CaCl_2_, CsCl and MgCl_2_. The self-assembly profiles are represented by the average cluster size. Snapshots of the aggregated disks at (**b**) stacked disks, (**c**) house of cards and (**d**) overlapping coin configurations.

**Figure 6:**
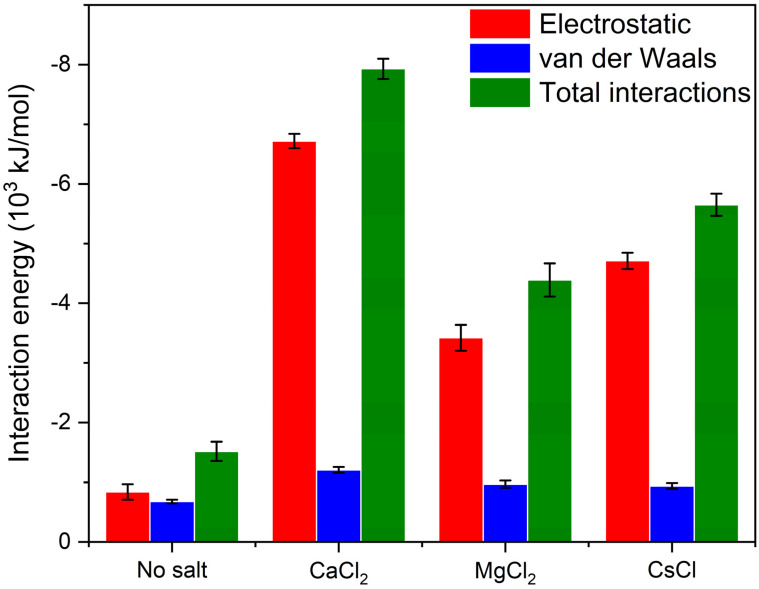
The average intermolecular interaction energies between the laponite disks in the absence and presence of 5 mM of CaCl_2_, MgCl_2_ and CsCl. The intermolecular interaction energies are the combinations of van der Waals and electrostatic interactions averaged over the last 5 ns of the simulation time. The error bars of the data represent standard deviations based on simulations performed in triplicate.

**Figure 7:**
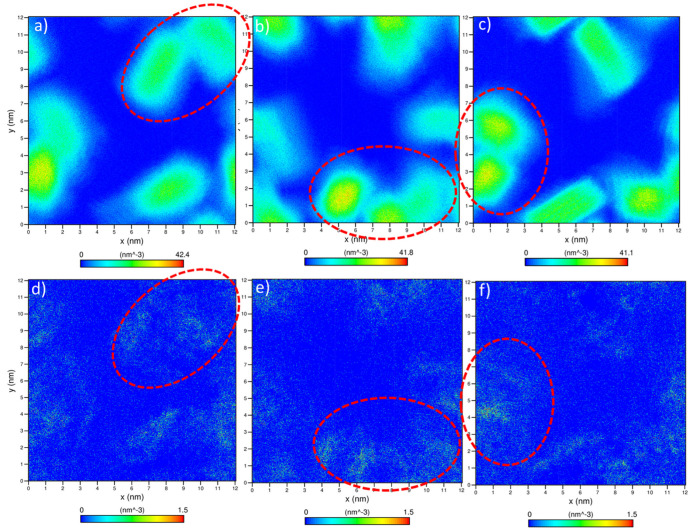
Density maps of the self-assembled laponite disks in the presence of (**a**) CaCl_2_, (**b**) MgCl_2_ and (**c**) CsCl and the accumulated (**d**) Ca^2+^, (**e**) Mg^2+^ and (**f**) Cs^+^ on the surface of self-assembled disks. In (**d**–**f**) representations, the laponite disks and the water molecules are not visible. The data are averaged over the last 5 ns of the simulation time. Periodic boundary conditions are applied to all density maps in x, y and z directions.

In the expression above, $N_{n}$ is the average cluster size and $N_{i}$ is the number of clusters containing *i* molecules. Two laponite disks are considered to be clustered if the distance between the disks was less than 3.5 Å based on the experimental separation distances between the aggregated disks in the presence of divalent cations [11]. Our data show that aggregation kinetics are fastest in CaCl_2_, followed by CsCl, MgCl_2_ and salt-free suspensions (Figure 5), which is consistent with the relaxation time $\tau_{Q}$ (Figure 3). All the laponite nanodisks are self-assembled into a single cluster in the suspensions bearing the salts. Smaller laponite clusters are noted in the salt-free suspensions (Figure 5a). The aggregated disks exhibited various configurations, namely overlapping coins, house of cards and stacked platelets, which agrees with the observations of Jonsson et al. [30] (see Figure 5b–d). The accelerated self-assembly of laponite suspensions is attributed to enhanced electrostatic interactions (Figure 6). The total intermolecular interactions (the combination of electrostatic and vdW interactions) changed from (−1.52 ± 0.13) 10^3^ kJ/mol in the salt-free laponite suspension to (−7.91 ± 0.12) 10^3^ kJ/mol, (−4.39 ± 0.22) 10^3^ kJ/mol and (−5.65 ± 0.14) 10^3^ kJ/mol in laponite suspensions prepared using CaCl_2_, MgCl_2_ and CsCl solutions, respectively. The uncertainties in the intermolecular interaction values represent the standard deviation based on simulations performed in triplicate. The total intermolecular interactions drive the changes in the aggregation behavior of laponite such that nanoparticles with stronger intermolecular interactions show higher aggregation numbers in shorter times. The faster laponite aggregation results in a slower diffusion coefficient, which is attributed to the fact that aggregates diffuse slower than individual nanoparticles and the diffusion coefficients follow the order of salt-free > MgCl_2_- > CsCl- > CaCl_2_-bearing suspensions. This dynamics behavior and the order of diffusion coefficients are consistent with the experimental relaxation time shown in Figure 3. The higher intermolecular interactions in the salt-incorporated suspensions are driven by the accumulation of the cations on the surface of the laponite disks to neutralize the negative charge (Figure 7). The higher cation densities noted between the self-assembled disks show that the intercalation of cations neutralizes the charges between the individual disks.

In these simulations, the chemistry and charge distribution at the face or “basal surface” differs from that of the rim or the “edge”. The face or “basal surfaces” are composed of a tetrahedral silicon layer which retains a negative charge while the rim is positively charged. Enhanced face-to-face orientations of the self-assembled laponite disks resulting from the accumulation of the cations are noted. Further, the anions do not preferentially accumulate around the positively charged disk rim (see Figure S2), which could be attributed to the small surface area of the rim, compared to the disc surface, of which requires few dispersed anions to neutralize its charge.

**Figure 8:**
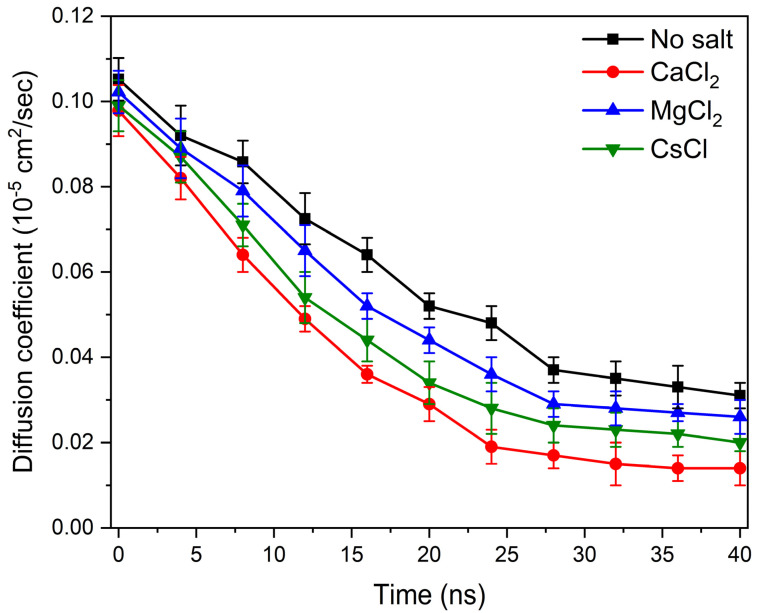
The self–diffusion coefficient of laponite suspensions determined from MD simulations as a function of the simulation time. The error bars of the data represent standard deviations based on simulations performed in triplicate.

The influence of the addition of salt on relaxation behavior is also evident from the time evolution of the diffusivities of the laponite nanodisks that are extracted from the trajectories of MD simulations (Figure 8). The self-diffusion coefficient (*D*) of laponite in the salt-free and salt-incorporated suspensions are calculated from the mean square displacement (*l*^2^), as follows:

(7) $$D=\frac{1}{6}\underset{t\to \infty }{lim}\frac{d\langle l^{2}\rangle }{dt}$$

As larger aggregates form, the mobility of laponite disks, as noted from the diffusivities, is considerably reduced. The diffusivity of the laponite suspensions of CaCl_2_-bearing solutions is considerably lower compared to the suspensions bearing CsCl and MgCl_2_ ions due to the formation of larger clusters, as noted in Figure 5. As the simulation time increased from 1 ns to 40 ns, the average self-diffusion coefficients of laponite nanodisks in salt-free suspension decreased from (0.105 ± 0.005) × 10^−5^ cm^2^/s to (0.031 ± 0.003) × 10^−5^ cm^2^/s, respectively. Similarly, the diffusion coefficients of CaCl_2_, MgCl_2_ and CsCl-bearing suspensions decreased from (0.097 ± 0.004) × 10^−5^ cm^2^/s to (0.014 ± 0.004) × 10^−5^ cm^2^/s, from (0.099 ± 0.006) × 10^−5^ cm^2^/s to (0.021 ± 0.002) × 10^−5^ cm^2^/s, and from (0.102 ± 0.005) × 10^−5^ cm^2^/s to (0.026 ± 0.004) × 10^−5^ cm^2^/s, respectively. This dynamic diffusion behavior is consistent with the evolution of the self-assembly of laponite suspensions. 

**Figure 9:**
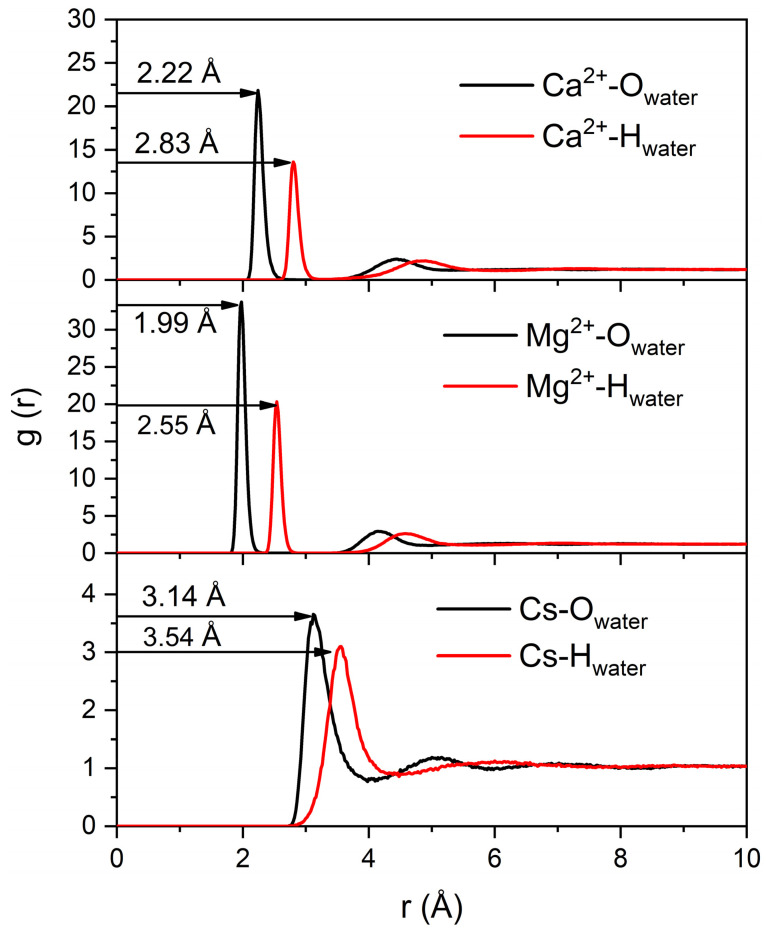
The radial distribution functions (RDF) of cation–water oxygen predicted from MD simulations in different laponite suspensions bearing (a) CaCl_2_, (b) MgCl_2_ and (c) CsCl salts.

It is interesting to note that the incorporated salts exhibit various hydration shell thicknesses and densities due to the interaction with individual laponite disks and aggregates (see Figure 9). The number of water molecules in the first hydration shell of Ca^2+^, where the relaxation is fastest, is higher in comparison to in Cs^+^ and Mg^2+^ cases. The number of water molecules in the hydration shells of Cs^+^, Mg^2+^ and Ca^2+^ are 5.8, 6.1 and 7.9, respectively, which is in agreement with previous studies [57]. The first peaks of RDF for the cation–oxygen atom of the water molecule are 2.22 Å, 1.99 Å and 3.14 Å for Ca^2+^, Mg^2+^ and Cs^+^, respectively, which is in agreement with previous studies [58,59]. The water molecules in the hydration shells are bonded to the ions, making it difficult to exchange with the bulk water. Thicker hydration shells result in the slower diffusion of the corresponding ions. Factors that contribute to the accumulation of ions with thicker hydration shells, such as Ca^2+^ and Mg^2+^ ions on the negatively charged laponite surfaces, include lower mobility resulting from the thick hydration shell and the electrostatic interactions with the laponite surface (Figure 7).

The main observation from the XPCS measurements and MD simulations is that the salt-bearing laponite suspensions reach an arrested state faster than the salt-free laponite suspensions. The higher electrostatic interactions in the laponite suspensions in the presence of salts contribute to an enhanced gel formation. Cations aid the charge stabilization of the laponite suspensions. In the absence of salt, the electrostatic interactions between the negatively charged surfaces and positively charged rims of laponite disks accelerate the formation of gels. In general, the cations with a higher charge and diameter have a greater influence on aging dynamics due to accumulation on the negatively charged laponite interfaces that dominate the surface area of the individual disks [7,11]. The atomic diameters of Mg^2+^, Ca^2+^ and Cs^+^ are 1.5 Å, 1.8 Å and 2.6 Å, respectively. The observed relaxation time of laponite suspensions is lowest in suspensions bearing Ca^2+^ ions followed by Cs^+^ and Mg^2+^ ions (Figure 3). The dominant influence of charge is evident from the effect of the Ca^2+^ ion, despite the smaller size compared to the Cs^+^ ion. The slower relaxation behavior of laponite suspensions in Mg^2+^ ions compared to Cs^+^ ions shows that the size of the cation dominates the charge in this scenario. These data suggest that both the ion radius and charge need to be considered when evaluating the influence of ions on the relaxation behavior of laponite suspensions.

## 4. Conclusions

In this study, the relaxation behavior of laponite suspensions and the influence of salts including CaCl_2_, MgCl_2_ and CsCl are studied using XPCS and MD simulations. The fluid-to-gel transformations of these suspensions are determined from the relaxation time obtained from the XPCS measurements. Competing and non-monotonic effects of charge and size of the cations on the relaxation behavior are noted. Ca^2+^ ions facilitate accelerated relaxation despite having a smaller cation size compared to Cs^+^. The relaxation times of laponite suspensions in the presence of Mg^2+^ ions are slower compared to Cs^+^ ions despite the higher charge, suggesting that cation size dominates in this scenario. Molecular dynamic simulations show the accumulation of cations at the surfaces of laponite and facilitate a faster self-assembly. The trends in relaxation behavior are consistent with the cluster formation behavior of laponite suspensions and the intermolecular interaction energies predicted from MD simulations. The charge balance is achieved by the intercalation of the cations at the negatively charged surfaces of laponite suspensions. These studies show that the arrested gel state of laponite suspensions is accelerated in the presence of salts and the competing effects of cation size and charge on the relaxation behavior of laponite suspensions. To achieve a better understanding, further studies are required to investigate the influence of salt concentration, aging time and other salt compositions. 

## Data Availability

All the data associated with this study is reported in the manuscript and the supporting information.

## Supplementary Materials

The following supporting information can be downloaded at: https://www.mdpi.com/article/10.3390/ma16010101/s1. The supplementary information file contains the size distribution of laponite nanoparticles obtained from SAXS measurements (Figure S1) and density maps of the dispersed anions (Figure S2).
