# Supplementary material for: Resolving Salt-Induced Agglomeration of Laponite Suspensions Using X-ray Photon Correlation Spectroscopy and Molecular Dynamics Simulations
Source: Materials (Basel). 2022 Dec 22;16(1):101. doi: 10.3390/ma16010101 (PMC9820912; doi:10.3390/ma16010101)
Supplement: Supplementary file 1 [file materials-16-00101-s001.zip › materials-2020328-supplementary.pdf]

Resolving Salt-Induced Agglomeration of Laponite Suspensions Using X-Ray Photon Correlation Spectroscopy and Molecular Dynamics Simulations

Sohaib Mohammed,[1] Meishen Liu,[1] Qingteng Zhang,[2]

Suresh Narayanan,[2] Fan Zhang,[3] and Greeshma Gadikota[1,]*

[1]School of Civil and Environmental Engineering, Cornell University, Ithaca, NY 14853, United States

[2]X-Ray Science Division, Advanced Photon Source, Argonne National Laboratory, Chicago, IL 60439

[3]Materials Measurement Science Division, National Institute of Standards and Technology, Gaithersburg, MD 20899

Corresponding Author. Phone: +1 607-255-4796. E-mail: gg464@cornell.edu

S1. Modeling the Scattering Curves of Suspended and Interfacial Laponite

The intensity curves *I(Q)* of laponite nanoparticles in salt-free suspensions describe the structure of scatters that are composed of the form factor [$P(\vec{Q})$] and the structure factor [$S(\vec{Q})$] such that:

$$I(Q) = n\left\langle P(\vec{Q})S(\vec{Q})\right\rangle \quad \text{(S1)}$$

In the equation above, *n* is a prefactor related to the number density of laponite nanoparticles. The form factor of the laponite nanoparticles reveals a disc-like structure with a diameter and thickness of about 34.67 nm and 1.08 nm, respectively **(see Figure S1)**.

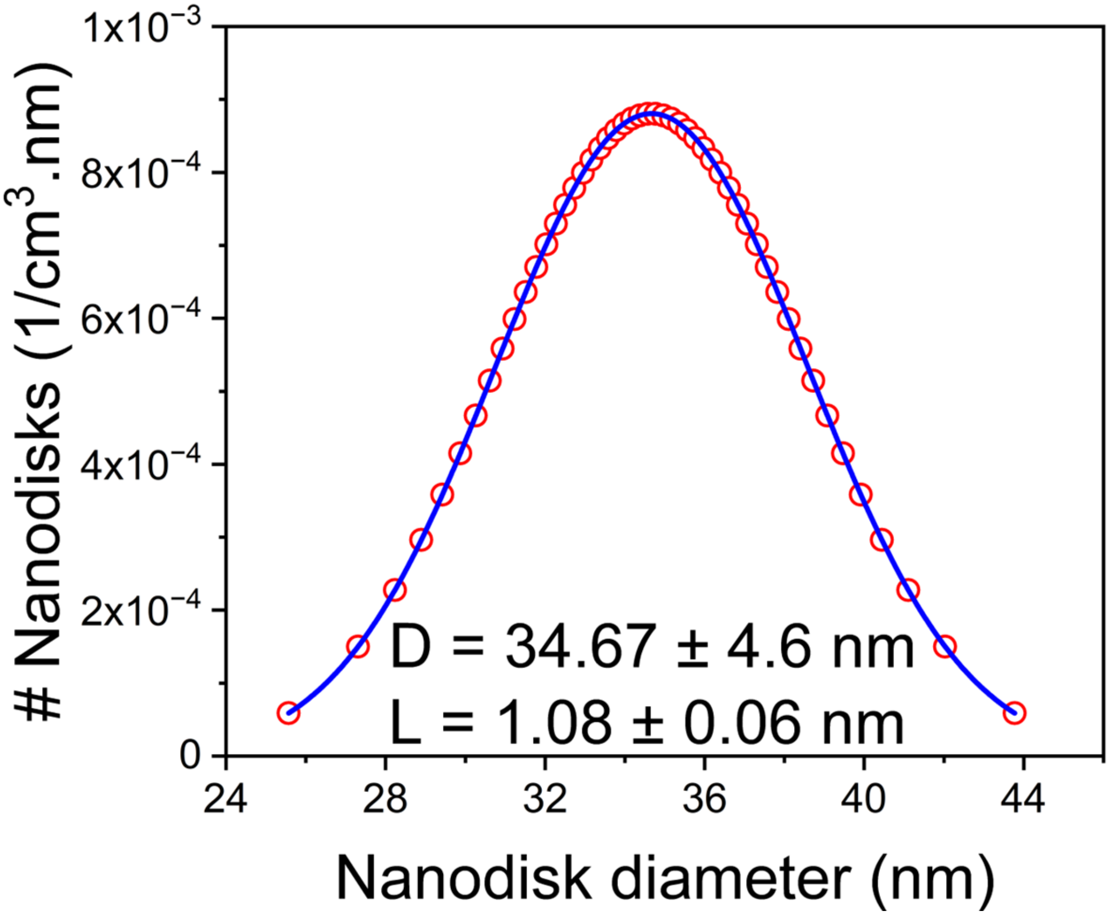

Figure S1. The number of laponite nanoparticles in salt-free suspensions as a function of the laponite nanodisc diameter.

S2. Anions positions in the equilibrated simulation cell

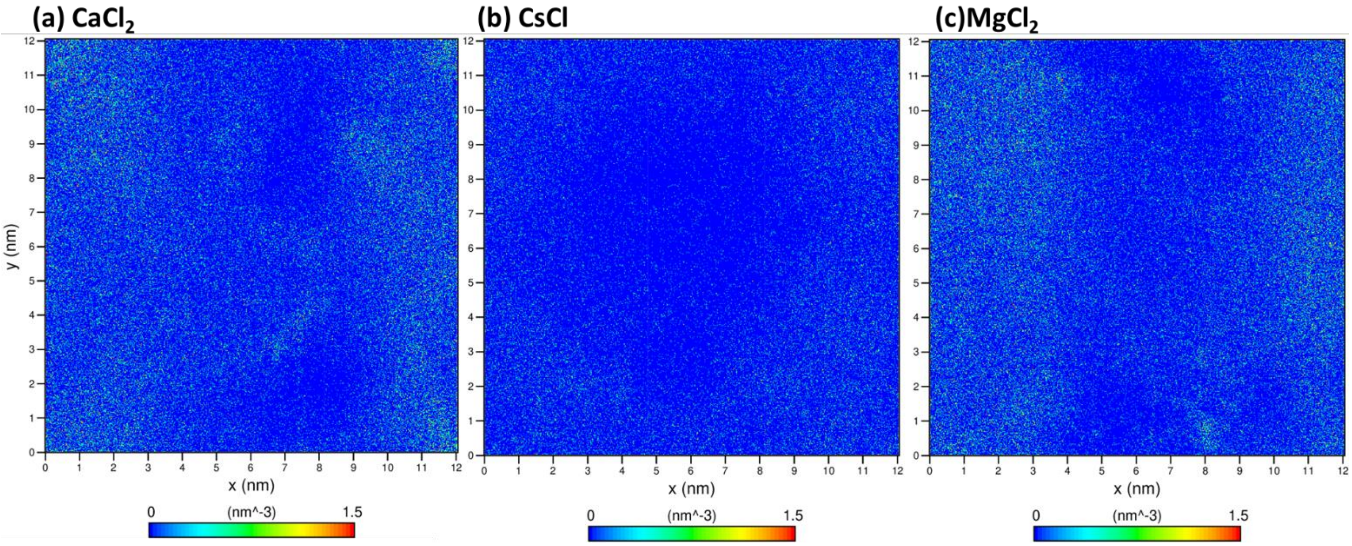

Figure S2. The density map of the anions averaged over the last 5 ns of the simulation time. Blue colors represent the low-density regions while green, yellow and red regions show the high-density regions.
